# Discourse developments within the public agenda on Danish nature management 2016–2021: Animal welfare ethics as a barrier to rewilding projects

**DOI:** 10.1007/s13280-023-01964-8

**Published:** 2023-12-09

**Authors:** Roland Vestergaard Kragh Christensen, Niclas Scott Bentsen

**Affiliations:** Department of Geosciences and Natural Resource Management, University of Copenhagen, Rolighedsvej 23, 1958 Frederiksberg C, Denmark

**Keywords:** Environmental discourse, Environmental policy administration, Machine learning, Natural language processing (nlp), Text mining

## Abstract

Prompted by the increasing public focus on environmental policy and the continuous inability of States to reach environmental targets agreed upon in the context of the United Nations and the European Union, we explore the development of discourses within the Danish public agenda regarding nature management 2016–2021. This is done through a mixed-methods framework of discourse analysis and structural topic modeling based on documents from the Danish Parliament’s Environmental committee 2016–2021, estimating topic prevalence, and analyzing the discourses within each topic, resulting in a qualitative overview of 21 identified topics and their associated discourses and an overview of how the different topic proportions changed over time. A shift in the public agenda was found: a change from discussions about untouched forest focused on trade-offs between timber extraction and biodiversity, to a discussion about different understandings of animal welfare in the context of large grazers in nature national parks in Denmark.

**Supplementary Information:**

The online version contains supplementary material available at 10.1007/s13280-023-01964-8.

## Introduction

### Development of international environmental policy

Environmental policy deals with preventing, reducing, and mitigating the harmful effects that human activities can have on nature and natural resources. The goal is to ensure that human interaction with the environment does not have harmful effects on humans or the environment (McCormick 2001). The concept of public environmental policy is known throughout history. Legal frameworks on sustainable land use and interactions with public goods such as water and air are observed in ancient Roman law (Sáry 2020). Similar instances are also present throughout the European Middle Ages (Tebrake 1988; Ostrom 1990). In modern times, the presence of environmental policy has been exemplified by the USA’s National Environmental Policy Act of 1969, which incorporated an assessment of environmental effects in federal decision-making (Anderson 2011). Through the United Nations Conference hosted in Stockholm, 1972, 26 principles on environment and development were internationally agreed on and The United Nations’ Environment program (UNEP) was established. This changed the nature of international environmental law from a bilateral framework to a global framework (Brunneé 2009). Especially principle 21, which establishes that States have the “responsibility to ensure that activities within their jurisdiction or control do not cause damage to the environment of other States” (United Nations 1972, p. 5), laid the groundwork for future international environmental law. However, international environmental law was still in a “mutual limitation paradigm” (Brunneé 2009, p. 3), where actual environmental policy was left for the individual States to define as long as it did not affect the neighboring States.

While the Convention on Biological Diversity (CBD) was praised for moving international environmental law into a more active paradigm by requiring states to take action, it was still criticized for its “soft, non-binding approach” (Arthur 1993, p. 10). Especially the use of ambiguous phrases such as “Each contracting Party shall, as far as possible and as appropriate” (ibid) was highlighted as points of critique. Targets for biodiversity conservation were specified in 2002 as the CBD adopted a Global Plant Conservation Strategy with 16 targets aimed to slow plant extinction by 2010 (Paton and Lughada 2011).

As a direct consequence of the UN’s Strategic Plan for Biodiversity 2011–2020, the EU’s Biodiversity Strategy 2011–2020 was formulated, which referenced the commitments the EU member states had made within the CBD Nagoya meeting in 2010 (European Union 2011). In 2020, the CBD concluded on the efforts of the UN’s Strategic Plan for Biodiversity, out of the 20 Aichi targets, none had been fully achieved and six targets had been partially achieved. Simultaneously, the EU Biodiversity Strategy 2011–2020 also failed to halt and reverse biodiversity loss by 2020 (European Commission 2022). In the evaluation of EU Biodiversity Strategy 2011–2020, the implementation of the strategy is assessed both on a general EU level and for each Member State (ibid). An interesting period of national environmental policy development is found in the State of Denmark 2014–2021.

### Danish public environmental policy 2014–2021

Denmark is governed through a multiparty system, where governments often are composed of coalitions or hold less than half of the 179 seats in parliament. Most law-making passes through committees specific to the given subject of the proposed law. Party members of all parties are present in every committee.

In the period 2014–2021, the public focus on environmental policy in Denmark saw a rapid increase. In 2014, Denmark adopted a national biodiversity strategy 2014–2020 (Miljøministeriet 2014), referencing the obligations made to the CBD in 2011, and the EU Biodiversity strategy 2011–2020. In 2016, the Danish conservative-liberal government party, Venstre passed a ‘Nature package’ (Miljø- og Fødevareministeriet 2016). The package addressed the targets of the EU Biodiversity strategy 2011–2020. Financially, the package earmarked 365.5 million DKK for a range of biodiversity initiatives, such as the goal of reaching a total area of 25,000 ha ‘biodiversity forest’ in Denmark. Biodiversity forest is a combination of 19,100 ha untouched forest and 5,900 ha forest where 15 trees are left as deadwood pr. harvested ha (ibid). The package was in effect 2016–2019.

The most mentioned topic during the Danish 2019 parliamentary election was climate and the environment (Blach-Ørsten et al. 2020). During the campaign, the Social democratic party and the Socialist people’s party proposed to ensure a total of 75,000 ha untouched forest and to establish 15 nature national parks (Socialdemokratiet 2018). Discussions on a new biodiversity package culminated in December 2020 with an agreement of a 'Biodiversity and nature package’ earmarking a total of 888 million DKK for untouched forest, nature national parks, creation of a biodiversity council, improvement of marine ecosystems, and an examination of the existing laws regarding nature and biodiversity to ensure a legislative framework capable of implementing the agreement (Miljøministeriet 2020). The ‘Biodiversity and nature package’ is in effect 2021–2024 and has a goal of reaching a total area of ca. 75,000 ha untouched forest and establishing 13 nature national parks (ibid).

### Insights in Danish environmental discourse

The shift in economic and spatial scale between the ‘Nature package’ of 2016 and the ‘Biodiversity and nature package’ of 2020 indicates a change in the public agenda regarding nature management in Denmark. Other studies also describe this change such as Stubager et al. (2020), which quantitatively tracks voters on a range of policy topics with questions like if the State is spending too much or too little resources on environmental issues. Similarly, Blach-Ørsten et al. (2020) tracked data from 99 news media through the Danish general election campaign period 2019 to measure how many times nine policy topics (EU, climate and environment, refugees and immigrants, welfare, economy, pension, health, adult-to-child ratio in daycare, and defense) were mentioned throughout the period. While these studies create an insightful overview of the focus on environmental policy in relation to other policy topics, their level of detail does not contain a qualitative insight in the way the Danish public speaks about the subject.

Such insights have previously been supplied by Læssøe et al. (2003), through 11 qualitative interviews with Danes of different backgrounds, the individual understandings of how people interact with nature were studied. Petersen (2007) studied a national discourse through news broadcastings during the 1992 UN summit in Rio and the 2002 UN summit in Johannesburg.

During the Danish general election of 2015, voters regarded environmental policy as the least important policy topic (Stubager et al. 2020). However, in the general election of 2019, it was seen as the most important topic. Further, the environment was the most mentioned topic by the media during the 2019 election (Blach-Ørsten et al. 2020). Although Denmark ratified the UN Strategic Plan for Biodiversity 2011–2020 and was committed through the EU Biodiversity Strategy 2011–2020, none of the strategic targets were achieved by 2020 (Secretariat of the Convention on Biological Diversity 2020, European Commission 2022). The contrast between public focus on environmental policy and failure to achieve the 2020 targets motivates an inquiry in the discourses of the public agenda on Danish nature management. Following the idea that “the way we speak about forests directly impacts their governance” (Leipold 2013, p. 12), an understanding of how the public speaks about Danish environmental policy might also result in an understanding the direction of Danish environmental policy.

This paper aims to identify the development of discourses within Danish public agenda regarding nature management 2016–2021 and to examine whether there has been a change in the public agenda. Discourse analysis is combined with topic modeling to identify topics within Danish public agenda on nature management, describe their related discourses, and understand the change of discourse prevalence over time.

Others successfully combine discourse analysis and topic modeling examining political discourses on crypto-market forums (Munksgaard and Demant 2016) and on contrasting perspectives on China during the 2019 Hong Kong protests (Stine and Agarwal 2020). Eranti et al. (2015) create a topic model based on the mention of climate change or global warming in The New York Times and The Hindu. Chawathe (2020) does something similar. However, combining discourse analysis with topic modeling has not been done for Danish environmental policy.

## Theoretical framework

### Discourse analysis

The field of environmental policy analysis hosts a variety of theoretical perspectives (Wandesforde-Smith 1974), one such theoretical perspective is discourse analysis, which focuses on the relationship between language and political action.

A discourse is defined as “an ensemble of ideas, concepts, and categorizations that is produced, reproduced, and transformed in a particular set of practices and through which meaning is given to physical and social realities” (Hajer 1993, p. 45). Here, the assumption is that different discourses “enable and constrain our ability to act on phenomena that are negotiated in environmental policy making” (Leipold et al. 2019, p. 447). Epistemologically, this school of thought is placed within constructivism, where it is assumed that our understanding of the world is repeatedly developed through social interactions which are contextualized by culture and history (Pedersen 2009; Ültanir 2012). In other words, through collective efforts of communication we define and redefine our understanding of the world (White 2004).

The study of discourses enables the examination of the formation and expression of truth claims and positioning for or against policy change (Leipold et al. 2019). Through the last 60 years, a theoretical and methodological framework for discourse analysis has been developed (Pedersen 2009), covering a broad field of approaches. Broadly, discourse analysis can be defined as “the study of social life, understood through analysis of language in its widest sense (including face-to-face talk, non-verbal interaction, images, symbols and documents)” (Shaw and Bailey 2009, p. 413). Discourse analysis is a broad field ranging from the individual elements of a discourse such as a speech, commercial, or painting to the connection between such elements and how they in relation to each other form a social structure.

Within the field, four general traditions of discourse analysis are present (Leipold et al. 2019):

1. Foucauldian, where discourse is a medium of power relations, as discourse defines and constrains our understanding of the world. Here, the analytical task is to examine how multiple discourses are produced to understand the underlying relation between power and knowledge.
2. The Habermasian tradition sees discourse analysis as a test of arguments. This is an idea of political argumentation as a deliberative practice, where it is the researcher’s task to examine the validity of different claims.
3. The critical discourse analysis is centered around the ambition to uncover the way discourses uphold and legitimize social inequalities.
4. The positivist approach seeks to incorporate an objective position for the analyst by examining socio-cultural structures through analysis of large sets of text data. Discourse analysis is not often applied as the sole framework for environmental discourse analysis, instead they serve as theoretical inspiration and are integrated into a mixed-methods design drawing on other frameworks (ibid).

### Representation and policy priorities: Are policymakers representative of the public?

We determine and measure the discourses of the Danish public agenda through archived questions and answers from the Environmental committee of the Danish Parliament. The opinions of policymakers are assumed to be inferable to the general public (Jones and Baumgartner 2004).

In a Danish context, Green-Pedersen and Wilkerson (2006) extended the research of Jones and Baumgartner (2004) by comparing political health care attention and policy development in Denmark and the US over fifty years. Danish political health care attention is measured through parliamentary debates and questions submitted by parliamentary members to a minister. Similar increased public attention to health care is found for both countries. In relation to the study at hand, Green-Pedersen and Wilkerson (2006) theoretically paves the way for using the connection between the public agenda and policymakers’ attention in a Danish setting.

### Topic modeling

Probabilistic topic modeling is an analytic approach developed within the field of machine learning. Topic modeling consists of a set of algorithms which statistically estimate how words are connected to documents, to each other, and how the relationships between words change over time (Blei 2012), thereby discovering latent themes called topics.

**Fig. 1:**
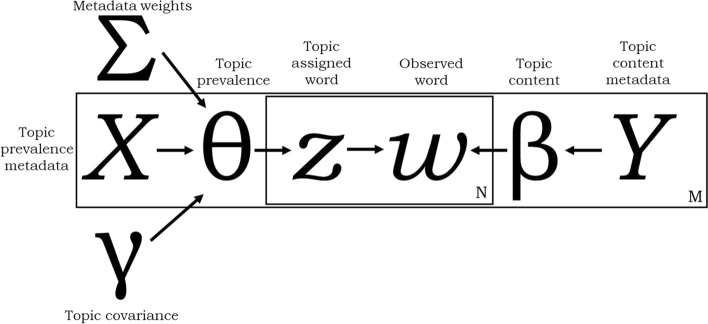
Simplified plate diagram of structural topic model process modified from Roberts et al. (2013)

The Latent Dirichlet Allocation (LDA) model proposed by Blei et al. (2003) assumes that topics, the order of documents, and terms are independent. The Correlated Topic Model (CTM) (Blei and Lafferty 2007) responds to the weakness and assumption of independence between documents by allowing for the modeling of correlation of latent topics. Lastly, the incorporation of metadata in topic modeling has been proposed as a need of the field (Blei 2012). Roberts et al. (2013) answer the call with the Structural Topic Model (STM), which allows for the incorporation of metadata as covariate to the model. In short, the left side of Fig. 1 calculates the proportion of words in a document which are attributable to each topic, providing a measure of topic prevalence. The right side calculates the words most likely to be generated by each topic, which provides a measure of the content.

## Materials and methods

### Research design

We employ structural topic modeling on a collection of questions asked and answered within the Environmental committee of the Danish parliament 2016–2021. The documents are selected based on a combination of search words designed to capture all documents regarding nature management. Nature management is understood in line with the World Bank’s definition of natural resource management as the “utilization of natural resources such as land, water, air, minerals, forests, fisheries, and wild flora and fauna” (World Bank 2001, p. 154). The quality of the structural topic model’s results is verified through qualitative coding of the ten most defining documents for each topic. Through the qualitative coding the discourses of each topic are assessed.

### Operationalization of discourse analysis

Public agenda is understood in line with Jones and Baumgartner (2004). Discourses are defined in line with Hajer (1993), and the broad definition of discourse analysis as the “analysis of language in its widest sense” (Shaw and Bailey 2009, p. 413).

The discourse analysis is carried out using coding, where words and phrases are annotated with a label, which categorizes thematic moments in a text (Hansen and Klemmensen 2014). The discourse analysis is inductive as it does not seek to test some previous understanding of how the data should be structured but seeks to explore and describe the structure which is thought to exist in the data.

### Operationalization of structural topic modeling

Structural topic modeling is carried out through the statistical computing and graphical environment, R. A package for structural topic modeling was published in 2014 and this analysis used version 7.9.11 from 2020.

Topics are latent themes in a corpus and are defined as “a mixture over words where each word has a probability of belonging to a topic” (Roberts et al. 2019). A corpus is a set of documents, and documents are bodies of text carrying meaning. In this study, a document is defined as a questioned asked and answered within the Environmental committee of the Danish parliament. A document is composed of a mixture of topics and the sum of the document topic proportions constitutes the topic proportions for a corpus.

### Combining discourse analysis and topic modeling

This study utilizes the ability of topic modeling to uncover the hidden semantic structures of corpuses through computation which otherwise would be very time consuming to assess manually (Blei 2012). The ability to process large datasets makes topic modeling a fit method for analyzing public policy processes such as parliamentary debates and more formal enquiries, where large collections of chronologically archived texts are available.

While the systematic approach of topic modeling can lift some of the practical barriers associated with discourse analysis such as human prejudice and the amount of time it takes to manually study text, it is critical to validate that the result of topic modeling constitutes meaningful units (Jacobs and Tschötschel 2019). A mixed-method pairing of topic modeling and qualitative discourse analysis is argued to be mutually beneficial (ibid). By combining the methods, it is possible to cover more empirical ground and ensure the validity of the analysis. The potential of doing so is noted and encouraged throughout the literature (Brookes and McEnery 2019; Jo 2019; Brinkmann 2019; Aranda et al. 2021).

### Limitation of the study

The public Danish nature management debate is a broad field, which in this study has been limited to questions raised in Danish parliamentary environmental committee. This has been done to simplify the data extraction process and size. Therefore, the study is engaged with a sample of the actors engaged in the field. The inferential potential to a general Danish context is introduced through Jones and Baumgartner (2004). In line with the research questions, the study is further limited to the period of 2016–2021.

### Selecting data

The questions and answers within the Environmental committee were accessed through the Parliament’s official website. Questions are always addressed to the environmental minister, and always answered by the minister and their administration. To specify documents regarding nature management, the website’s search engine which accepts Boolean operators was utilized with the search words: Biodiverse OR nature national park OR “untouched forest” OR forest OR “nature and biodiversity package” OR nature protection OR nature restoration OR “nature” OR Nature management.^1^ The final set of search words were selected through an iterative process. The combination of search words which produced the least topics not related to nature management was chosen. Discarded words include Area, region, and nature without the specifying quotation marks.^2^

A weakness of topic models is ‘polysemy,’ where words can have several meanings depending on their contextual use (Jacobs and Tschötschel 2019). The same can be said for metaphors, aphorisms, wordplays, or other types of language where the meaning is understood through context. Topic modeling is unable to determine if a word was used sincerely or ironic. However, the use of questions and answers from the committee mitigates this weakness by having certain standards for permitted language use. In the handbook for parliamentary work for members of the Danish parliament, it is stated that committee questions must be formulated ‘neutral’ and without value-laden expressions (Folketinget 2022a).

### Data collection

The documents were gathered using a web scraper. A scrape of the results page of the search words resulted in 2058 documents. Each document contained repetitive formal information in the beginning such as “The Parliament’s Secretariat for Law^3^,” which administrative worker who presented the answer, and the address of the environmental ministry. This information was removed as best as possible. A command ensuring that all documents were unique was run.

**Fig. 2:**
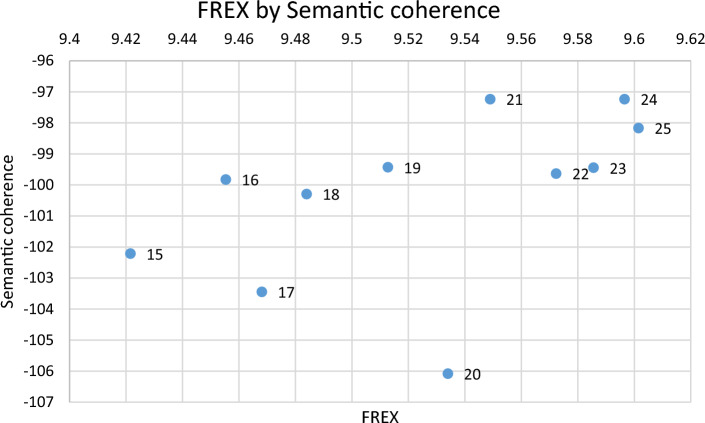
The FREX score by semantic coherence score estimated for models with 15–25 topics

The final dataset contained 2176 rows and ran from August 15th 2016 to December 9th 2021, with a single document from July 1st 2022 (Fig. 2).

### Selecting the number of topics

The number of topics is “a function of your research question” (Stewart 2017), therefore the number of topics must accommodate a meaningful overview of the topics’ content, but also a certain degree of nuance and uniqueness within each topic.

Two diagnostic numeric values can aid the selection of topic numbers, semantic coherence, which “is maximized when the most probable words in a given topic frequently co-occur together” (Roberts et al. 2019, p. 11), and frequent exclusivity (FREX), which is calculated as the weighted harmonic mean of a word’s rank regarding exclusivity and frequency. FREX is a measure of how much a word only appears in one topic. Numerically, the ‘best’ semantic coherence score is found closest to 0, and the ‘best’ FREX score is the highest possible score. The combination of these goodness of fit measures provides a trade-off which must be balanced to select the number of topics (Roberts et al. 2019).

A set of criteria for the selection of the number of topics were put together in the following hierarchical order:

1. The number of topics must be of a size which reduces manual coding of sampled documents for each topic within the project timeframe but also without degrading the topic quality substantially.
2. Semantic coherence and FREX scores are used in combination to evaluate topic quality of a given K.

Manual coding of more than 25 topics was deemed impossible. The exclusion of more than 25 topics might distort the nuances of the topics a bit but aids the general overview of the data. Models with less than 15 topics were also excluded as these were found to consist of many overlapping topics. Based on the above criteria 21 topics were selected (Fig. 2).

To validate the quality and content of the topics, the ten most defining documents for each topic were sampled for discourse analysis. Some topics contained duplicates, and to compensate, an extra document was gathered for each duplicate. Additional duplicates collected through this process were not compensated, as the sample size should allow for discourses to emerge.

### Correlation test

A correlation test was carried out between the 21 topics to explore their quantitative relationship. This allowed for another check of whether the topics were overlapping, something which is also addressed in the selection of the number of topics, and through the qualitative coding. If two or more topics are strongly correlating it might indicate that they are about the same theme. The correlation test employs a 0.05 cutoff correlations coefficient value to capture some of the negligible correlation coefficients which exist between 0.0 and 0.1. Further, weak correlation coefficients exist between 0.1 and 0.39 (Schober et al. 2018).

### Qualitative coding

The qualitative data analysis used the software package, Nvivo. The coding process was structured through 21 top codes, one for each topic, and a sub-code for each word identified by the stm within the given topic. The subcodes were composed of the seven highest scoring semantic coherence and FREX words for each topic. If the same word was present both in the semantic coherence and FREX list, it was merged and only coded once. The finished coding product is an overview of all 21 topics, their subcodes, and all text related to the identified words. The subcodes were used to create an overview of all text where the different topic words were present. This gave an understanding of how different topic words interacted and how they were used to formulate different discourses. For each topic, the overall theme of the topic and the quality of the topic characterized as either high, medium, or low were stated. A high-quality topic is composed of a clearly defined theme. Medium quality is achieved if the topic holds two or three clearly defined themes. Low quality is achieved if no clear theme is identified.

## Results

### Discourses present in the Danish nature management debate 2016–2021

The qualitative analysis of the 21 topics found six topics of low quality, six of medium quality, and nine of high quality. Table 1 grants an overview and brief description of all topics. A more elaborate analysis of the 21 topics is found in the Supplementary Information.

**Table 1:** A table of the topic numbers, their highest probability and FREX words, assigned topic title, and topic quality

No.	Highest probability words	Topic label	Topic quality
	FREX words		
1	Municipality, the municipalities, the municipality, the administration, submitted, along, informs	Wild boars and African swine fever	Medium
	Swine fever, injunction, african, wild boar, along, municipality, silver salmon		
2	Agriculture, fisheries, the agency for agriculture, subsidies, organic, support, the scheme	Subsidies grazing of nature areas	High
	The agency for agriculture, the scheme, commitment, the farmers, subsidy, paid out, payment		
3	The nature conservation law, the nature conservation law’s, the rules, paths, roads, travel, access	Change of nature conservation law and expropriation	Medium
	Fertilization, spraying, conversion, protected, the prohibition, prohibition, expropriation		
4	Law, coastal protection, amendment, proposal, permit, connection, concrete	Coastal protection permit	High
	Coastal protection, simplification, game management, vvm, division of competences, environmental assessment, programs		
5	University, isthmus, aarhus, the goal, the agriculture, climate, nitrogen	Reduction of nitrogen discharge and agriculture	High
	Isthmus, the goal, gudenåen, eelgrass, climate, watershed plan, pressure factors		
6	Forest, untouched, forests, trees, afforestation, the agreement, areas	Biodiversity and untouched forest	High
	Untouched, tree, forest area, trees, forest, afforestation, biodiversity forest		
7	Watercourse, law, strong, proposal, municipalities, water, designation	Reassessment of watercourses and the EU Water Frame Directive	High
	Water courses, water councils, artificial, highly, modified, which, water planning		
8	Denmark’s, the committee, proposal, nature conservation association, comment, the inquiry, between	Nature conservation boards	Low
	Nature conservation association, the conservation board, meeting, conservation cases, organizations, the committee, the conservation boards		
9	Law, animal, nature national parks, animal husbandry, change, the animals, proposal	Animal welfare and nature national parks	High
	The nature national parks, the animal welfare law, the animals, the traffic law, road safety, category, animals		
10	Nitrogen, substances, condition, discharge, phosphorus, good, tons	Discharge of nitrogen and other hazardous substances	High
	Waste water, phosphorus, discharge, environmentally hazardous, mapping, treatment plant, nitrogen		
11	Species, university, data, danish, nature types, dce, aarhus	Red listed species	High
	Red list, red-listed, species, conservation status, dce, center, threatened		
12	Nature, areas, biodiversity, nature, the areas, natural areas, more	Biodiversity goals	Medium
	Natural areas, biodiversity, biodiversity, state-owned, the areas, nature, natura areas		
13	Natural, dispensation, hearing, areas, proposals, the beach protection line, new	Natura 2000 hearings	Medium
	The beach protection line, consultation response, consultation, wind turbines, the danish Energy Agency, dispensation, natura		
14	Requirements, rules, application, nature, regulation, companies, used companies, products, requirements, the requirements, regulated, keep, the regulation	Regulation of different trash and ammonia	Low
	Requirements, rules, application, nature, regulation, companies, used companies, products, requirements, the requirements, regulated, keep, the regulation		
15	Mink, pollution, dumping, assessment, submitted, surface water, substances mink, burial, patting, surface water, taken out, cheminova, the regions	Pollution from Cheminova, buried mink, and dumping of construction sediments	Medium
	Mink, pollution, patting, assessment, submitted, surface water, substances mink, burial, patting, surface water, taken out, cheminova, the regions		
16	Aquaculture, establishment, law, compensatory, means of action, marine, expansion	Aqua culture and kelp facilities	Medium
	The aquaculture, salmon lice, compensatory, means of action, environmental protection, kelp facility, mariculture		
17	Million, annually, submitted, funds, discloses, costs, set aside	Budgeting phrases	Low
	Million, set aside, payment, costs, the national parks, sales, funds		
18	Areas, protected, natura, designation, protection, protected, hunting	10% strictly protected EU areas	High
	Strictly, protected (definite plural), protected, bird protection areas, designated, -areas, areas		
19	Groundwater, data, pesticides, the groundwater, the answer, the period, food product	Pesticide contamination of groundwater	Low
	Pesticides, geus, groundwater, groundwater, findings, the agricultural package, food products		
20	Fishing, danish (plural), regulation, tools, agriculture, bottom trawling, danish (singular)	Fishery regulation	Low
	Tools, bottom trawling, the seabed, and, the, bottom trawl, vessels		
21	The rules, access, the nature protection law’s, covered, nature protection law, regulations, land	Public access and signage	Low
	The nature protection law, the nature protection law's, the rules, paths, roads, travel, access		

### Shift of discourse prevalence from 2016 to 2021

**Fig. 3:**
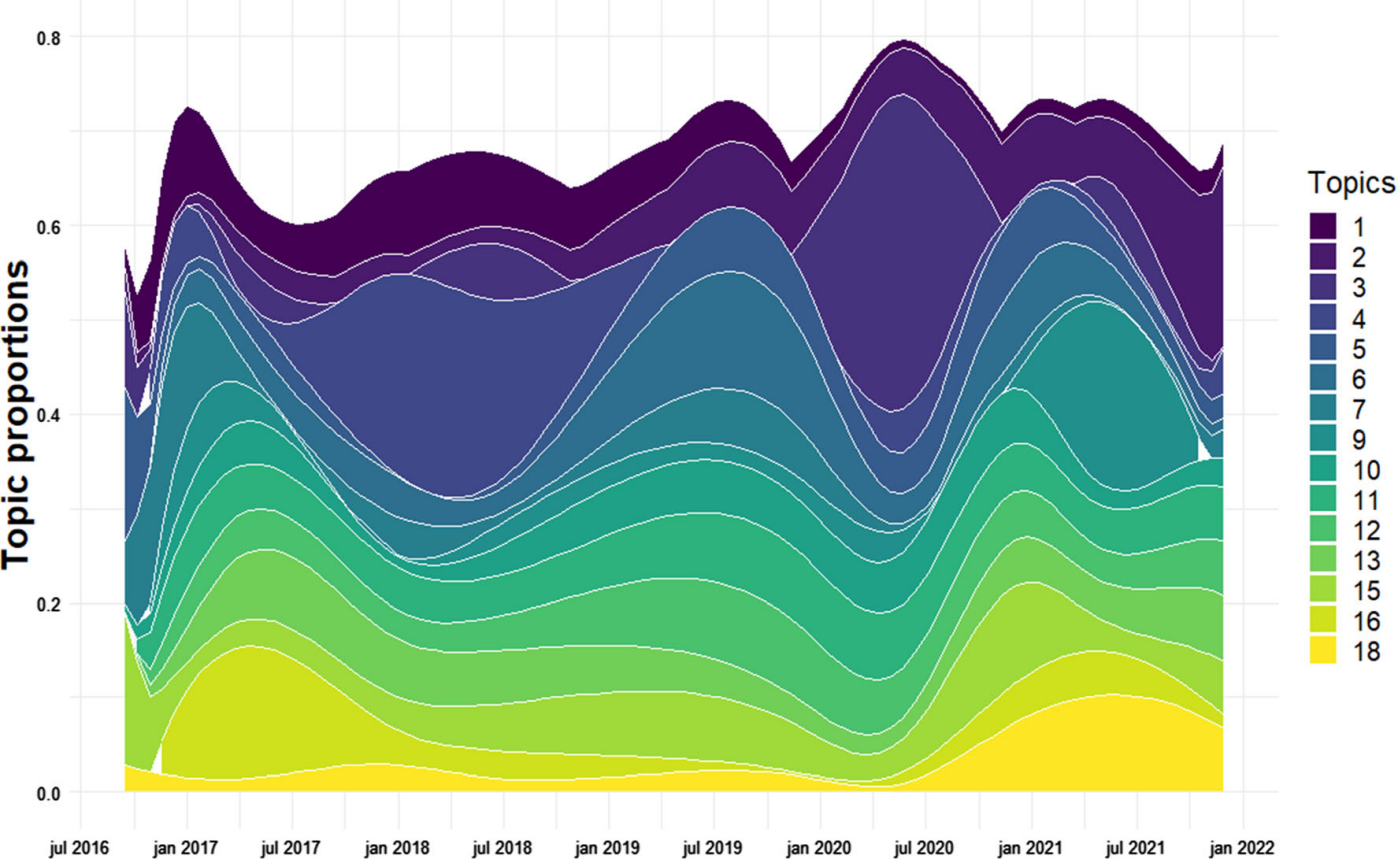
Stacked area plot of topic proportions 2016–2021

The analysis of topic proportions excluded the low-quality topics. The remaining 15 topics were plotted as a stacked area plot both as the topic proportion values over time (Fig. 3). As the total topic proportion is always summed to 1, the topic proportion values show how much of the total topic proportion was discarded with the lower quality topics.

The topic proportions shows that some topics, such as Red listed species (topic 11), Biodiversity goals (topic 12), Natura 2000 hearings (topic 13), Pollution from Cheminova, buried mink, and dumping of construction sediments (topic 15) are quite stable over time, while others fluctuate and hold a large proportion at some points and close to none at other points of time. This phenomenon is especially found for Change of nature conservation law and expropriation (topic 3), Coastal protection permit (topic 4), and Animal welfare and nature national parks (topic 9). As an example, both the highest and lowest topic proportion are found for Change of nature conservation law and expropriation (topic 3), with a value of 0.1% the January 30th 2016, and 33.3% May 22nd 2020.

**Fig. 4:**
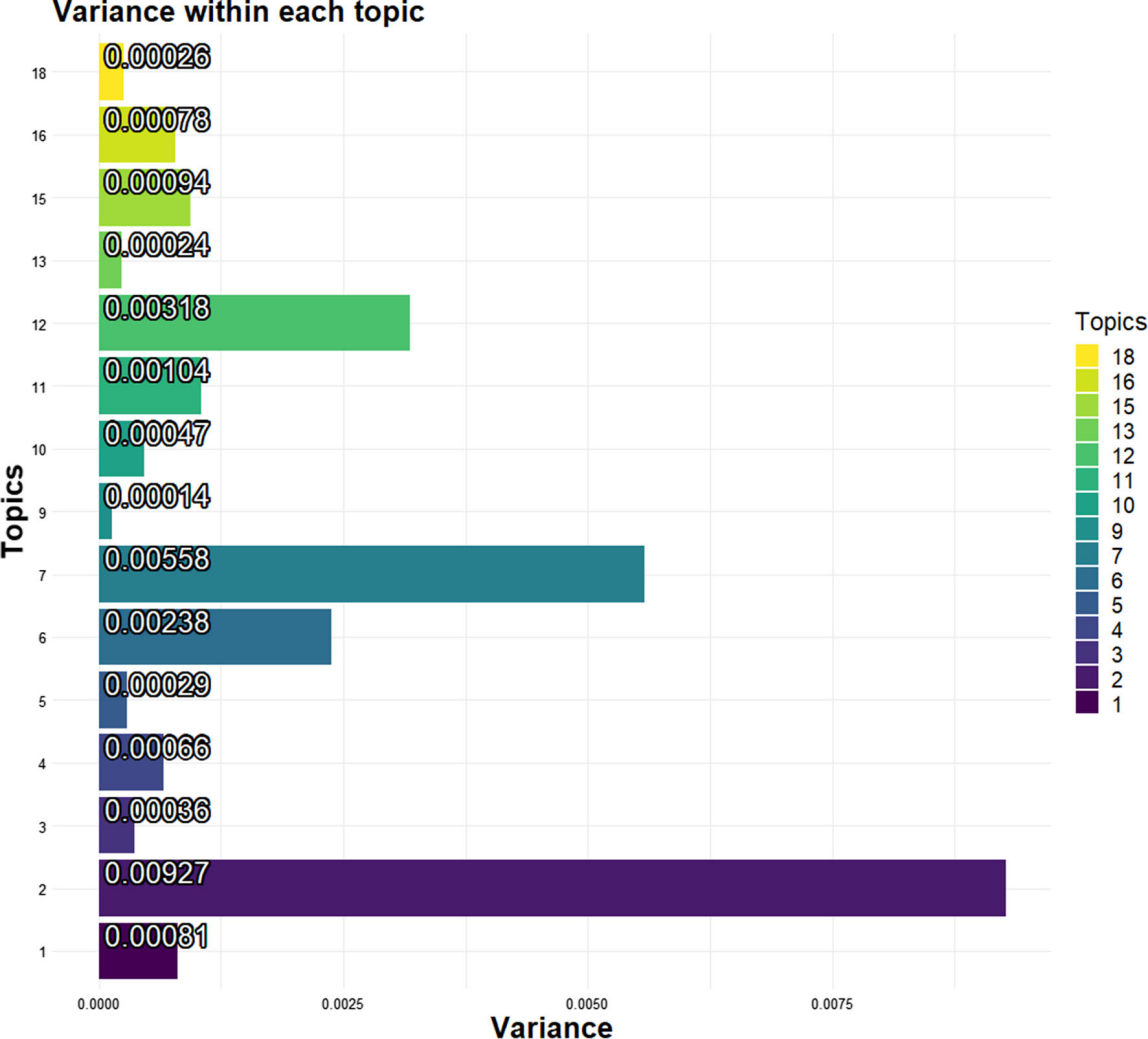
Variance within each topic

Change of nature conservation law and expropriation (topic 3) and Coastal protection permit (topic 4) are confirmed as the topics with most variance (Fig. 4). Opposite, Red listed species (topic 11) and Discharge of nitrogen and other hazardous substances (topic 10) are the topics with least variance. None of the topics have a variance of 0, meaning that all underwent some change over time.

**Fig. 5:**
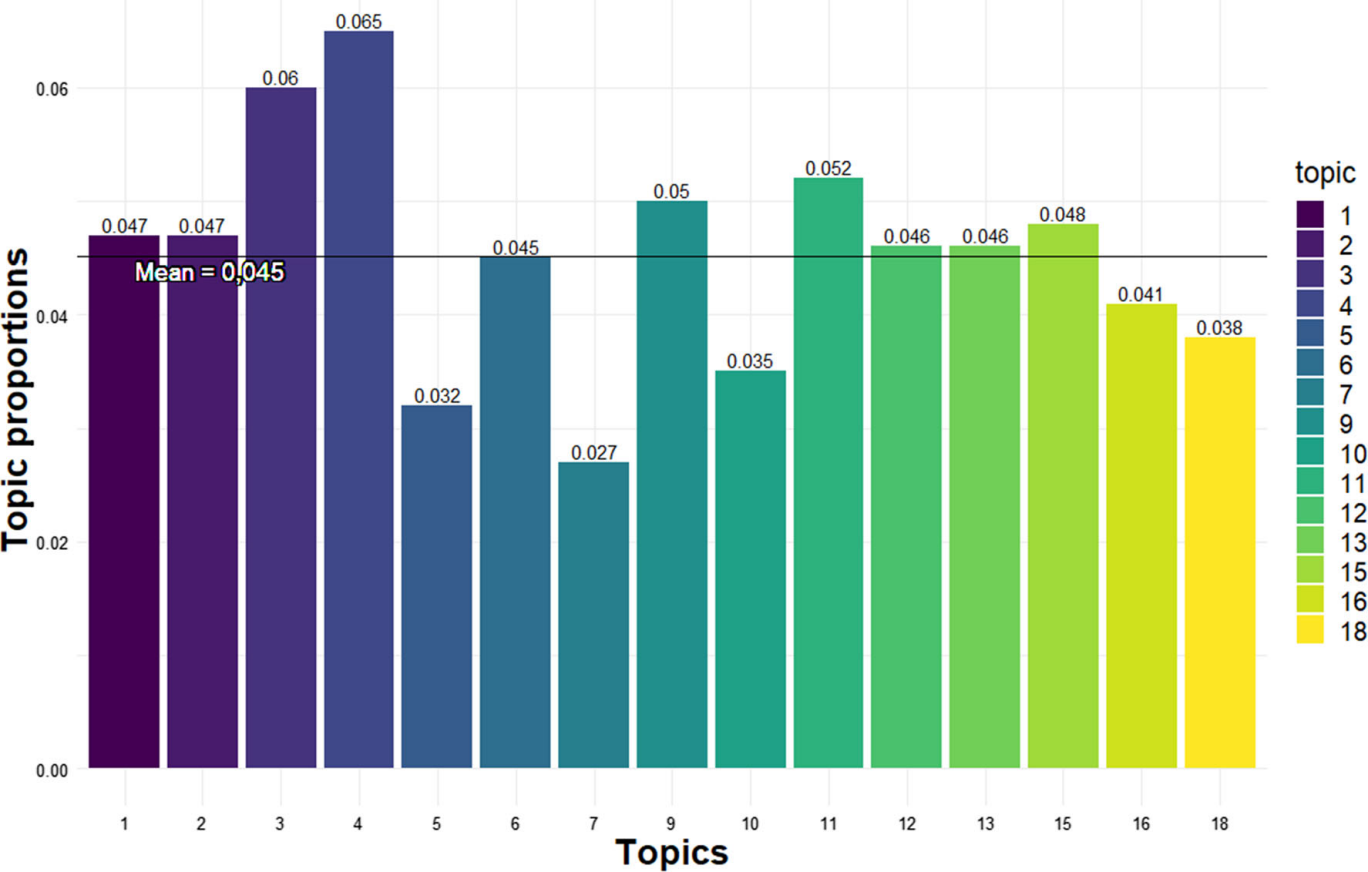
Mean topic proportion for 2016–2021

The mean topic proportions show that Change of nature conservation law and expropriation (topic 3) and Coastal protection permit (topic 4) had the highest mean proportion at 0.06 and 0.065, respectively (Fig. 5). The overall mean topic proportion was 0.045. Nine of the topics exists over the mean and six are found under the mean.

### Topic correlation

The correlation test showed a 0.12 correlation between Reduction of nitrogen discharge and agriculture (topic 5) and Discharge of nitrogen and other hazardous substances (topic 10). A correlation ‘chain’ was found between Biodiversity and untouched forest (topic 6), Biodiversity goals (topic 12), Public access and signage (topic 21), Natura 2000 hearings (topic 13), and 10% strictly protected EU areas (topic 18).

### Negative values

Out of 1350 topic proportion values, 63 negative values were observed. Recalling that topic proportions exist between 0 and 1, it is theoretically impossible for a topic to exist less than 0. Negative topic proportions mainly exist in the beginning of the dataset and around July 2019. The most extreme negative values seem to be a product of lacking data. The lack of data in the beginning of the dataset is a result of the Danish Parliament’s summer break which last from June to October, during which no committee work is scheduled. The lack of data around July 2019 can be explained by the Danish general election which was declared the 26th of May 2019 and new government was formed the 27th 2019 of June. During an election all committee work is halted for the politicians to focus on the election campaign (Folketinget 2022b). Based on this, the negative values were removed from the data.

## Discussion

### Did the prevalence of discourses change?

Quantitatively the question of whether the prevalence of discourses shifted from 2016 to 2020 can partly be answered through the variance for the topics. A change was detected within all topics, some topics seem to be quite stable over time and others show a ‘spiked’ behavior of sudden large increases in proportion followed by sudden decrease.

The spiked topics are in some cases a product of a public agenda and an overestimation caused by the law-making structure. While there is congruence between policy makers’ and the public agenda, policy makers split issues in smaller units than the public to effectively attend the different issues (Jones and Baumgartner 2004). This seems also to be the case for this topic model, where the spikes are policy makers attending larger underlying topics. A spike unrelated to a specific law is found in Biodiversity and untouched forest (topic 6), showing that not all spikes reflect restricted periods of time in which politicians can discuss a certain topic.

### Which discourses were present in the Danish nature management debate?

The described discourses paint a picture of Danish nature management debate which on one hand calls for an increase of the strictly protected nature areas and criticizes the lack of Danish biodiversity and progress regarding internationally pledges, but on the other hand a concern that increased nature protection limits possibilities for economic growth and nature experiences like hunting and fishing is also raised. Another detected change of prevalence is from UN-focused Biodiversity goals (topic 12) to the EU-focused 10% strictly protected EU areas (topic 18). Showing a change of focus in the Danish public agenda from the international environmental commitments made on an UN level to an EU level.

### A change of tone

A rather substantial change seems to be found between the two topics qualitatively connected to the themes of the nature packages: Biodiversity and untouched forest (topic 6) and Animal welfare and nature national parks (topic 9). As they touch on the two main themes from the packages: Untouched forest and nature national parks with large grazers.

**Fig. 6:**
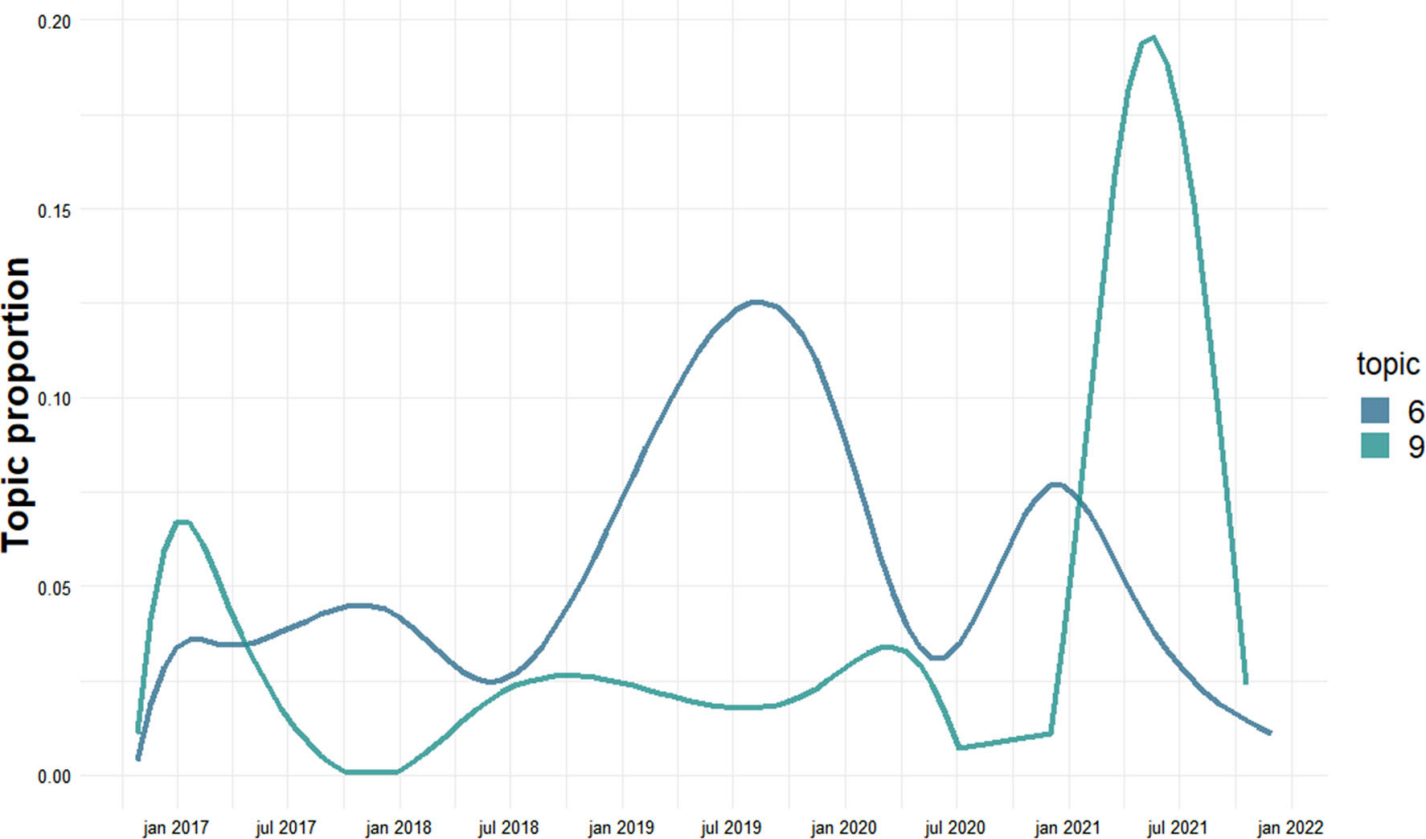
Topic proportion for biodiversity and untouched forest (topic 6) and animal welfare and nature national parks (topic 9)

The highest peak in Biodiversity and untouched forest (topic 6) is a topic proportion of 0.13 (Fig. 6) during July 2019, at the end of the first nature package 2016–2019, which aimed at declaring a fifth of all State forest “biodiversity forest.” In a Danish understanding, untouched forest is defined as an area where commercial forestry has ceased with the goal of favoring biodiversity (Danish Nature Agency 1992). In recent times, actions such as restoring natural hydrology, variations in stand structures, and the removal of non-native species are a part of the initial management of newly declared areas (Danish Nature Agency 2021). The qualitative results for topic 6 show a concern for continued timber harvest in areas declared to become untouched forest. The quantitative increase in topic proportion suggests that the consequences of the 2016–2019 package are observed and discussed at the end of the package, decreases to a previous topic level, and then increases as the next government starts discussing the nature and biodiversity package 2021–2024.

The Nature and biodiversity package of 2021–2024 introduced an increased focus on grazing as a nature management tool, especially in regard to the proposed ‘nature national parks.’ Animal welfare and nature national parks (topic 9) shows a small increase in the focus on grazing around January 2017, which might be explained by the “proposal of law regarding amendment of environmental approval etc. of livestock, environmental protection law, agricultural use of fertilizers and plant cover law and various other laws” which was proposed the January 12th 2017, and then a rapid increase again in 2021 as the Nature and biodiversity package 2021–2024 was discussed and established. Here, the peak topic proportion was 0.17.

The differences in time between the topic proportions for Biodiversity and untouched forest (topic 6) and Animal welfare and nature national parks (topic 9) show that the discussion of untouched forest is followed by a discussion of animal welfare. In regard to the declaration of untouched forests and the nature national parks, a voice is raised against continued harvest of timber in the State’s forest, as it is seen as damaging and hindering to the planned increase of biodiversity though untouched forest. On the other hand, the negative consequence that untouched forests will have on the supply of timber for national industry is also represented. Denmark is ca. 15% forest; the State owns ca. 18% of the forest. In contrast, Finnish and Swedish forest cover is ca. 70% and Norwegian is 40% (Ekström and Hannerz 2021).

Round the run of the century, the Danish land use plan was to double the cover of forest by year 2100 and increase the national timber supply from 20 to 45% (Tvedt et al. 2000). The change to a complete stop of commercial forestry on the State’s areas therefore proposes a sharp change of course for the Danish forest and timber industry.

While untouched forest is discussed as an economical and societal matter, restoring ecology through less monitored grazers is discussed as an ethical dispute of the definition of animal welfare. The difference between untouched forest and grazers is that the declaration of untouched forest is commonly agreed on as a trade-off between ecosystem services, where provisioning services are less prioritized. This trade-off is open for compensation through negotiation. However, animal welfare seems to be a more ethical topic, where the conflict stems from a disagreement on how animal welfare is defined. This ethical focus is harder to compensate through concessions within or outside of the nature management policy arena. Therefore, a substantial change in the Danish public agenda on nature management 2016–2021 is from a debate on untouched forest to animal welfare in nature national parks.

### Animal welfare: A new post-environmental position

Through analysis of Danish television coverage of the 1992 UN summit in Rio and the 2002 UN summit in Johannesburg, Petersen (2007) finds that the public Danish discourse on the environment in 1992 saw environmental disasters as a result of human actions and the solution as limited growth. This view transformed into an understanding of environmental problems as something where the solution is compatible with development of profitable businesses, leaving behind the need for limited growth. In 2002, an overarching economic discourse had been established, seeing environmental solutions as the opposite to economic growth.

A similar economic discourse is identified in 2016–2021. In Reduction of nitrogen discharge and agriculture (topic 5), where reduction of nitrogen discharge is criticized as damaging to the employment rate and the production of goods, and the minister notes that “the goal is not to produce less, but to produce smarter,” showing an unwillingness towards limited growth as a mean of environmental action. Petersen (2007) uses the term ‘post-environmentalist’ to describe this type of discourse, where environmental claims are criticized from another, in this case economic perspective. The public discourse of 2002 is described taking a post-environmental turn.

The presence of a post-environmental discourse is further supported by Stubager et al. (2020), reporting a decline in the proportion of voters who thinks that environmental actions should not negatively influence the private businesses from 1990 to 2007. The trend changes from 32% in 2007 to 55% in 2011.

An environmental discourse is present in topics such as Biodiversity and untouched forest (topic 6), where a discourse is that biodiversity should be prioritized over timber extraction. Biodiversity goals (topic 12) shows a discourse calling for Denmark to reach the pledged Aichi targets. Within Aqua culture and kelp facilities (topic 16), the environmental risks of salmon aquaculture are raised, here economic development is criticized from an environmental position. These examples show that the public agenda 2016–2021 consists of both environmental and post-environmental discourses.

The environmental counter to the post-environmental economic position is also observed in Stubager et al. (2020), finding that voters favor an increase in public spending over decreased private business revenue when it comes to environmental actions, and an increasing group either agrees or are indifferent with a reduction of private business as a consequence of environmental actions.

While Petersen (2007) finds economic development as a post-environmental position in 2002, a new animal welfare post-environmental position is identified within the Animal welfare and nature national parks (topic 9). Here, the nature management practice of imitating natural grazing dynamics through less regulated grazers is criticized as animal cruelty. The presence of a post-environmental animal welfare position is further supported by the Danish government’s visions material from 2022. It proposes to revert the change in the animal welfare law which gave a dispensation for reduced surveillance of animals in nature national parks, so that “the animal welfare in the nature national parks is ensured” (Regeringen 2022, p. 34), showing that the Government accommodates this post-environmental animal welfare position.

In the Netherlands, the case of Oostvaardersplassen (OPV) faced a similar post-environmental critic. Here, the management choice was to cull approximately a third of the population of large grazers, which in turn resulted in negative national and EU attention, forcing the project to be redrawn as a recreational national park, with less focus on natural grazing dynamics (Kopnina et al. 2019). The Danish rewilding project of 2016, Molslaboratoriet, pledges that an overpopulation of grazers is not handled by culling, but by giving animals to other nature projects. While the Danish project has met resistance, it continues to operate. Molslaboratoriet distances itself from OPV by monitoring the welfare of the animals and by not allowing its grazers to die naturally. Severely sick or injured animals are euthanized. Intentionally letting grazers of nature national parks die of natural causes seems to be outside the tolerance of the general public (Sandøe et al. 2022). For short-term management it should be considered how the lack of carcasses in a ‘rewilded’ area can be mimicked. Is it tolerable for the public if euthanized grazers are buried or kept out of sight? Long-term policy development should consider the migration of gray wolves into Denmark. What will the public opinion be of projects with fenced in wild grazers easily accessible to a pack of wolves? Both at OPV and Molslaboratoriet vigilantes have fed the animals to prevent animal cruelty. Wolves in Denmark have previously been illegally killed (Sunde et al. 2021), and civil disobedience aimed at protecting grazers from wolves might evolve. The wolf colonization of Germany has happened through military training areas, mainly due to the low anthropogenic disturbances (Reinhardt et al. 2019). In a similar Danish situation, should the state seek to prevent wolf migration in line with the pro-farmer sentiment of Wild boars and African swine fever (topic 1), assist the migrating by helping the gray wolf cross to the islands, or act indifferent?

### Topic correlations

Correlations found between the topics do not cause any concern for the analysis. Quantitatively, most of the correlations are placed in the shift between “negligible correlation” and “weak correlation” just around a coefficient of 0.1. The low correlation coefficients indicate that should an overlap between the topics be present, it is minor and should have no effect on the overall distinctiveness of each topic.

### Duplicates

Increased detection of duplicates will lower the uncertainty of overestimation that the duplicated documents impose. As the web scraper initially found 2058 unique urls, and the final data frame held 2176 documents, 118 extra documents are present in the data. This means that ca. 5% of the data are composed of duplicates of different types and represents a slight overestimation which is evened out between topics.

### Other types of data

Parliamentary data were initially chosen due to its public availability. However, data from newspapers have previously been used to track family policy in Germany (Gülzau 2019) and could potentially also have been used regarding Danish nature management policy. Transcribed news broadcasts and televised debates could also have been used. Including media data could help fill out some discursive holes in the dataset, as the news media outlets are a central part of the political systems’ function and are on their own a political institution (Albæk and de Vreese 2010). As a representative data source, media data might reveal opinions not present in the discourse of the politicians and the ministry, and thereby increase the understanding of the public opinions on nature management. For an overview of the political alignment of news media outlets from the perspective of citizens see Schrøder et al. (2022), for an overview of the interface between media and politics in Denmark see Nørgaard Kristensen and Blach-Ørsten (2020), and for an overview from the perspective of journalists see Skovsgaard et al. (2012).

Another type of parliamentary data which could have been used is the parliamentary debates and §20 questions. Green-Pedersen and Wilkerson (2006) used both parliamentary debates and committee questions in their study of health care attention and policy development in Denmark. Adding more parliamentary data might help even out topics which are centered around the legal treatment of a single law.

### Further research

Methodological, this study adds to the literature on structural topic modeling which is a relatively young method within topic modeling. Adding to the volume of a mixed-method analysis provides the opportunity of further discussing and developing the method. Further this analysis introduces a unique dataset, which tests the capabilities of structural topic modeling on an unexplored topic.

Other forms of metadata could be introduced to further explore the data. Using the political party as covariate could explore how different parties talk about the same topic and which topics parties are inclined to speak about. A combination of time and political party could explore how different parties talk about a topic over time and how their discourse changes. Broader classifications could be created on larger political structures like left and right wing or opposition and government. Inspired by Geese (2020) who explored which factors that make legislators talk about immigration in parliamentary debates, the speech actions of individual policy makers could be examined using a set of metadata such as party membership, age, voting district, and how many years they have been in the parliament.

## Conclusion

Utilizing the questions and answers from the Danish Environmental committee 2016–2021, a dataset of 2176 documents containing political opinions on nature management was compiled. Through a mixed-methods framework of structural topic modeling and discourse analysis, the text of the documents was divided into 21 topics, the proportions of topics were estimated over time, and the discourses related to the topics were described.

The change of topic prevalence was captured by plotting their proportions over time. The knowledge of the topics’ discourses and their proportions over time were used to describe how the Danish public agenda on nature management 2016–2021 has moved its focus from Biodiversity and untouched forest (topic 6) to Animal welfare and nature national parks (topic 9). In relation to discourses this meant a shift from a discussion on the ecosystem services trade-offs related to establishment of untouched forest to a discussion of how animal welfare is defined. The implication of this shift is that policy makers and nature managers have to consider solutions agreeable to the broad public.

This study showed the potential of combining discourse analysis with structural topic modeling, as it allowed for the analysis of large quantities of text at a qualitative level. It further established an introductory overview of the discourses present in the Danish nature management debate 2016–2021. Further research can either build on the structural topic model, adding more metadata and other types of documents carrying discourses or it can use the overview as a way of identifying and framing in-depth studies of one or more discourses present in the Danish nature management debate 2016–2021.

### Supplementary Information

Below is the link to the electronic supplementary material.

Supplementary file1 (PDF 242 kb)

## Footnotes

1 Translated from the Danish searchwords: biodivers OR Naturnationalpark OR “Urørt skov” OR skov OR “Natur- og Biodiversitetspakke” OR naturbeskyttelse OR Naturgenopretning OR “Natur” OR Naturforvaltning.

2 Translated from Danish: areal, område, and natur.

3 Translated from Danish: Folketingets Lovsekretariat.
