# Supplementary material for: Discourse developments within the public agenda on Danish nature management 2016–2021: Animal welfare ethics as a barrier to rewilding projects
Source: Ambio. 2023 Dec 9;53(4):637–52. doi: 10.1007/s13280-023-01964-8 (PMC10920536; doi:10.1007/s13280-023-01964-8)
Supplement: Supplementary file 1 — Supplementary file1 (PDF 242 kb) [file 13280_2023_1964_MOESM1_ESM.pdf]

Ambio

Supplementary Information.

This supplementary information has not been peer reviewed.

Title: **Discourse developments within the public agenda on Danish nature management 2016-2021: Animal welfare ethics as a barrier to rewilding projects**

Authors: Roland Vestergaard Kragh Christensen & Niclas Scott Bentsen

Qualitative description and assessment of each of the 21 topics. Each topic is denoted by its topic number, title reflecting the content of the topic, and the topic quality (low, medium, high).

Topic 1 – Wild boars and African swine fever and water plan - Medium

The topic is mainly about the risk of wild boars crossing the southern border of Jutland and introducing African swine fever to the domestic pork production. The topic quality is medium. In relation to a boar fence along the Danish-German border, there exists a technical understanding of the fence as a remedy to stop infected boars from crossing the border. Further a pro EU and anti-EU discourse is detected, as the fence is seen as a symbol of isolating Denmark from the rest of the EU (Schultheis, 2019). A lesser part of the topic is related to the municipalities' implementation of the state's water plan. Here a technical discourse is found in the inquiries regarding the status of the municipalities creation of injunctions prohibiting treatment of wastewater from remote settlements.

The documents regarding wild boars and municipal wastewater plans share the administrative words "submitted" and "informs", which might explain how the algorithm clustered the two themes.

Topic 2 – Subsidies grazing of nature areas – High

This topic is about delayed payment of subsidies to farmers who aid the management of nature areas where grazing is required. The overall quality of the topic is high.

A discourse negative of the administration's handling of subsidies is detected, as it is asked why the money is delayed, how the minister is going to pay out the money as fast as possible, and when the money will be received by the farmers. An opposing discourse is from the minister and the administration stating that the delayed subsidies are less important than the implementation of a new it-system, which in the long run will make the process better and more efficient. The topic word, "fishery" exclusively appeared with "agriculture" in the structure of the "Ministry of food, agriculture, and fishery". Another string of words appearing together is "subsidies", "paid out", and "payment".

Topic 3 – Change of nature conservation law and expropriation - Medium

This topic is formed by the proposal of "Law regarding amendment of the nature conservation law (prohibition of spraying, fertilization, and conversion of §3-protected areas)" which is perceived to have an element of expropriation of private farmland. The overall quality of the topic is medium. The phrasing of the law makes up a large portion of the topic words. The topic revolves around the consequence of the law for property owners: Will the consequence be a situation of "expropriation", where the state has to pay a dispensation, or a compensation-free regulation where the state does not have to pay a dispensation? The main discourse is that there are no general criteria which dictates whether an area will be seen as expropriated or compensation-free. The minister and

administration's discourse is that each assessment of an area is based on the specific circumstances. The topic words "paths", "roads", "travel", "access", and "the rules" were not detected in the sample indicating a possible second topic.

Topic 4 – Coastal protection permit - High

This topic is defined by the wording "Proposal of law regarding amendment of the coastal protection law, environmental assessment of plans and programs and concrete projects (vvm) law, nature protection law, hunting and game management law, and other laws (new division of competences and simplification of case processing regarding coastal protection etc.)", all documents in the sample is on the topic of "coastal protection" and "permit", which refers to the changes proposed by the law. The quality of the topic is high. The law seeks to concentrate the administrative competencies regarding permits for coastal protection at a municipal level. The questions are mainly on the topic of providing the municipalities and citizens with resources that help them navigate the new laws. Some question the benefits of the law change. All topic words are found in the sample. Most was identified in the title of each document which is the name of the law. "coastal protection" and "permit" was also found frequently through the text indicating a clearly defined topic.

Topic 5 – Reduction of nitrogen discharge and agriculture - High

This topic is centred around reduction of nitrate discharge and how this will affect the Danish agriculture. The topic quality is high. The reduction of nitrate discharge is called for by the EU and by the Danish water area plan 2021-2027. The questions are centred around two themes: The consequences reduced nitrate discharge will have on the employment and profit of agriculture. The minister's answer to this type of question is that "the goal is not to produce less, but to produce smarter". The second type of question is related to calculations on reference states, target nitrate discharge load, and a statement from Aarhus university which calls for a reduction of farmland by 500,000 ha. The questions are focused on whether the nitrate discharge targets are politically or academically motivated. The ministry ensures that the targets are based on academic work. It is also questioned why the reference states for nitrate discharge are changed from measurements made in the 1900's to a hypothetical "untouched" point of time. The ministry informs that this is in line with EU policy and that the measurements from the 1900's might already be influenced by nitrate discharge.

Two discourses are present in this topic. A discourse following the EU guidelines on nitrate discharge reduction, which is supported by the technical knowledge from Aarhus University. The second discourse is pro agricultural status quo, which questions whether nitrate discharge reduction should be prioritized over higher employment and production of goods and questions the legitimacy of the new ways of measuring nitrate discharge.

Topic 6 – biodiversity and untouched forest - High

This topic is about the nature packages of 2016 and 2019, specifically the untouched forest aspects of them. The quality of the topic is high. Half of the sample is composed of

questions regarding if and why old trees and broadleaves trees in areas which might be declared as biodiversity forest or defined as an “untouched area”. The ministry generally replies that timber harvest is a result of long-term planning and that it is a very small percentage of the harvest that is more than 80 years old and broadleaf. The discourse among the questions is that the ministry should harvest even less trees to protect and enhance the biodiversity of the state forests. Other questions are on the different rates at which broadleaves and conifers sequestrate carbon-dioxide, the budgets for creating “biodiversity forest”, and how untouched and biodiversity forest will have a negative effect on consumers’ ability to use wood for construction instead of plastic, cement, and steel.

Topic 7 - Reassessment of watercourses and the Eu Water Frame Directive - High

This topic is formed by the “Proposal of law regarding amendment of water planning law and other laws (incorporation of municipalities and water councils in the demarcation of water courses in water area plans and qualification of designation of artificial and highly modified water courses and new organization in the environmental- and food resources ministry”. The topic is about the classifications of the Danish water courses being updated as a consequence of the implementation of EU’s Water Frame Directive. The topic quality is high. The inquiries to the ministry are about clarifications of the criteria in the EU’s Water Frame Directive, to which degree more water courses will be designated as heavily modified during the reassessment, and what “nature value” means in regard to “water course”. The nature value is rated based on three parameters: Fish, plants, and small animals. Further structural properties are also included in the assessment. A technical discourse seeking clarification of the legal changes and technical details is detected. Many of the ministry answers simply refer to the answer given to Question nr. 60, which was not included in the sample.

Topic 8 – Nature conservation boards – Low

This topic is generally about the administrative case work carried out by nature conservation boards. However, two of the documents do not hold any of the topic words, two documents are speech notes for the minister (a document type which the data processing was meant to filter out), and three documents simply hold the reply that the ministry did not have the sufficient time to formulate a reply. The topic quality is low. Based on the documents which hold topic words, the main theme of the topic is questions regarding the how much time the administrative work of the nature conservations boards takes, and who applies areas for nature conservation, which is the Danish nature conservation association in most cases.

Topic 9 – Animal welfare and nature national parks - High

This topic is based on the “Proposal of law regarding amendment of nature protection law, law about forests, animal welfare law, law about field and road peace (access to establish nature national parks and mandatory digital communication, etc.)”. In this topic the consequences of dispensation from some of animal welfare requirements for animals in nature national parks and “rewilding” experiments is inquired. The topic quality is high. The

main dividing discourse line in this topic is whether animals can be supervised on a population level and still receive the care and attention that the Animal welfare law prescribes. The questions carry the discourse that this is not possible since hurt animals naturally would hide and therefore not be assessed. Further the discourse critical of the law change carries that it is not possible to assess the individual animals' layer of fat. A critical tone regarding 'rewilding' and the use of 'wild' animals is detected in this discourse. The ministry does, on the other hand, state that the individual animals' welfare is not lowered by the law change, and that grazing animals are essential tools for creating nature value in nature national parks. The overall discourse of the ministry is that the amended law will not lower the animal welfare.

Topic 10 – Discharge of nitrogen and other hazardous substances - High

This topic is about discharge of environmentally hazardous substances in Denmark. The topic quality is high. Nitrogen is the most mentioned topic word, which is used in three general themes for the topic: Aqua culture, wastewater treatment and spillage, and the general discharge level of Denmark over time. A technical discourse seeking clarification is observed in the questions. This is also exemplified through the topic words "good" and "condition", which are used to inquire about a given area's condition.

Topic 11 – Red listed species - High

This topic is about red listed species in Denmark. The topic quality is high. A third of the sampled questions are critical of the methodology behind the Danish red listed species 2019, stating that there has been a positive development of the red listed species and asking if the Danish method diverts from the IUCN guidelines in different ways, as an example "Can the minister confirm that the creation of the red list 2019 has not followed IUCN's guidelines?". The topic quality is high. There is a split in discourse where the questionnaires are critical of the results of the Danish red listed species 2019 and the environmental minister who states that the work of Aarhus university and the administration is correct. The rest of the questions are of technical matter regarding species populations in different areas.

Topic 12 – Biodiversity goals – Medium

This topic is about biodiversity and nature in the state's areas. Seven of the sampled documents are asking the minister for a response in the light of either public criticism of Denmark's performance regarding biodiversity, or for a status on Denmark's progress with internationally pledged goals such as Aichi. Two of the questions are of a technical character, and one is about the future of agriculture on the island, Livø. The topic quality is medium. The overall discourse of the questions is that there is not being done enough to reach the targets that Denmark has internationally pledged to. The Minister's response is that it is the responsibility of the united EU biodiversity strategy, which Denmark is a part of, deflecting the responsibility towards EU.

Topic 13 – Natura 2000 hearings - Medium

This topic is about the hearings of proposals for new and adjusted Natura 2000 areas. The topic quality is medium. The sample documents are questions of a technical discourse regarding upcoming and concluded hearings, such as how much new Natura 2000 area that is being considered, in which municipalities they are proposed, the number of hearing answers etc. Four topic words were not found in the data: Dispensation, the Danish Energy Agency, coastal protection, and wind turbines. Which indicates a possible second topic about coastal wind turbines.

Topic 14 – Regulation of different trash and ammonia - Low

This topic is about regulation of ammonia and methane pollution from farm animals, single use plastic products, regulation of littering from cigarettes, and regulation of chemical outdoor tile-cleaner. The topic is constructed of many small debates on trash and its effect on society. The ammonia and methane sub-topic make it hard to designate an overall topic, as does the nature of the different trash-subjects. Therefore, the topic quality is low as it is constructed of too many documents with different themes. The topic word "products" is the most occurring, it is found in relation to "plastic", "tobacco", "cleaning agents", and "producers", possibly explaining why the different types of trash was pulled together in a topic.

Topic 15 – Pollution from Cheminova, buried mink, and dumping of construction sediments - Medium

The sampled documents are mainly on the topic of pollution stemming from the company Cheminova, both newly detected pollution and future management of known contaminated sites. Other documents the culled mink buried as a part of the Danish Covid response. The burial sites are deemed a contamination risk for nearby water bodies. Lastly the word "dumping" which describes the practice of removing a top layer of fine sediments during construction projects on water and dumping the sediments at sea. No documents mentioning dumping was a part of the sample, but it is reasonable to believe that three topics are present. The topic quality is medium. A technical discourse asking about the details regarding polluted areas is detected. Another discourse is on the responsibility of the polluted areas, and if the Environmental agency is to blame for the lack of pollution stopping actions from Cheminova. In regard to the buried mink a discourse critical of the selection of burial sites is present in the questions. The topic word "surface water" is found within Cheminova and mink documents, tying them together as both forms of pollution are a risk to surface water.

Topic 16 – Aqua culture and kelp facilities - Medium

This topic is about compensatory marine means actions in relation to the establishment of aquaculture. The topic quality is medium, as two discourses are identified in the documents. A scepticism of expanded salmon aquaculture due to the risk of salmon lice, and a critical tone of the bureaucratic processes for establishment of kelp facilities, which is a new field of

business. Kelp is mentioned as a compensating marine means of action for soaking the increased nutrition levels related to aquaculture. This is the reason the two topics have been paired.

Topic 17 – Budgeting phrases - Low

This topic is constructed of yearly budgeting phrases, such as "10 million set aside yearly". Further the topic word "submitted" is related to the procedural phrasing "I have submitted the question to the Coastal agency". The topic quality is low.

Topic 18 – 10% strictly protected EU areas - High

This topic is about the EU biodiversity strategy where 30% of the total EU area is to be designated as protected and 10% as strictly protected. The topic quality is high. Two discourses are identified, on one hand there is a concern that hunting and fishing will not be allowed in the strictly protected areas and that the laws applying to protected and strictly protected areas are written in the EU-parliament and not the Danish. On the other hand, there is a concern that Denmark should at least designate 10% of its areas as strictly protected.

Topic 19 – Pesticide contamination of groundwater – Low

This topic is about a new set of data submitted by GEUS which informs that the level of pesticide contamination of the groundwater is higher than previously assumed. The topic quality is low, as the topic is constructed of two briefs, and two duplicated questions, where one holds no answer, and lastly a question on the statistics of dogs being put down due to biting.

Topic 20 – Fishery regulation – Medium

This topic is mainly about regulation of fishery, one document is about political coordination between different committees. Two discourses are detected regarding fishery regulation: Bottom trawling should be limited or stopped due to the damage it does on the seabed. The regulation of fishery does not follow the advice of scientific reports in the sense that fishery of clams should be increased. The topic quality is medium. The topic word "agriculture" is from the "the Minister of food, agriculture, and fishery" and is present in both the fishery topic and the political coordination.

Topic 21 Public access and signage - Low

This topic is about public access to privately owned nature areas and the public's right to establish signage in open areas such as fields. The topic quality is low as there are several documents which are not about public access or signage. The topic word "access" seems to be the culprit of error as it is used to carry the meaning of physical access to an area but also the administrations access to a range of legal actions. Further, the different version of the topic word "nature protection law" is found commonly throughout most documents.
